# Sociotechnical Intervention for Improved Delivery of Preventive Cardiovascular Care to Rural Communities: Participatory Design Approach

**DOI:** 10.2196/27333

**Published:** 2022-08-22

**Authors:** Michelle Partogi, Simon Gaviria-Valencia, Mateo Alzate Aguirre, Nancy J Pick, Huzefa M Bhopalwala, Barbara A Barry, Vinod C Kaggal, Christopher G Scott, Maya E Kessler, Matthew M Moore, Jay D Mitchell, Rajeev Chaudhry, Robert P Bonacci, Adelaide M Arruda-Olson

**Affiliations:** 1 Mayo Clinic Rochester, MN United States

**Keywords:** sociotechnical, secondary prevention, atherosclerotic cardiovascular diseases, community health, rural health, participatory design, team-based care

## Abstract

**Background:**

Clinical practice guidelines recommend antiplatelet and statin therapies as well as blood pressure control and tobacco cessation for secondary prevention in patients with established atherosclerotic cardiovascular diseases (ASCVDs). However, these strategies for risk modification are underused, especially in rural communities. Moreover, resources to support the delivery of preventive care to rural patients are fewer than those for their urban counterparts. Transformative interventions for the delivery of tailored preventive cardiovascular care to rural patients are needed.

**Objective:**

A multidisciplinary team developed a rural-specific, team-based model of care intervention assisted by clinical decision support (CDS) technology using participatory design in a sociotechnical conceptual framework. The model of care intervention included redesigned workflows and a novel CDS technology for the coordination and delivery of guideline recommendations by primary care teams in a rural clinic.

**Methods:**

The design of the model of care intervention comprised 3 phases: problem identification, experimentation, and testing. Input from team members (n=35) required 150 hours, including observations of clinical encounters, provider workshops, and interviews with patients and health care professionals. The intervention was prototyped, iteratively refined, and tested with user feedback. In a 3-month pilot trial, 369 patients with ASCVDs were randomized into the control or intervention arm.

**Results:**

New workflows and a novel CDS tool were created to identify patients with ASCVDs who had gaps in preventive care and assign the right care team member for delivery of tailored recommendations. During the pilot, the intervention prototype was iteratively refined and tested. The pilot demonstrated feasibility for successful implementation of the sociotechnical intervention as the proportion of patients who had encounters with advanced practice providers (nurse practitioners and physician assistants), pharmacists, or tobacco cessation coaches for the delivery of guideline recommendations in the intervention arm was greater than that in the control arm.

**Conclusions:**

Participatory design and a sociotechnical conceptual framework enabled the development of a rural-specific, team-based model of care intervention assisted by CDS technology for the transformation of preventive health care delivery for ASCVDs.

## Introduction

### Background

Atherosclerotic cardiovascular diseases (ASCVDs) are the leading cause of morbidity and mortality in the United States and exemplify the national urban-rural health disparity [1,2]. Rural populations, which comprise 20% of the US population, have a 40% higher absolute prevalence of ASCVDs than urban dwellers [2]. According to the American Heart Association (AHA), rural residents also have higher rates of uncontrolled cardiovascular risk factors than their urban counterparts [2]. These risk factors include tobacco use, hypertension, and high cholesterol [3-6]. For patients with established ASCVDs, adherence to risk modification strategies prevents adverse events, improves survival, reduces the need for revascularization procedures, and enhances life quality [7]. However, strategies for risk modification are underused by patients with ASCVDs, especially in rural communities [2,8].

Clinical practice guidelines from the AHA and the American College of Cardiology for patients with ASCVDs recommend risk modification strategies, including antiplatelet and statin therapies, blood pressure control, and cessation of the use of tobacco products for secondary prevention in patients with established ASCVDs [7,9-11]. These recommendations are collectively referred to as cardiovascular guideline recommendations *(V4)* and are also endorsed by the “Million Hearts” initiative from the Centers for Disease Control and Prevention [12]. The *V4* recommendations have been designated as Class I, which indicates that the supporting data are strong and treatment is useful and effective and should be administered to most patients under most circumstances [9,13]. The level of evidence that supports these guideline-endorsed recommendations is also considered to be of the highest quality (level of evidence designation “A”) as it is derived from multiple randomized controlled trials [9,14,15].

### Objectives

Multiple health care system factors affect the appropriate delivery of cardiovascular risk modification strategies to rural residents [2]. One factor is the inadequate number of physician providers in rural communities, as documented by the World Health Organization, the AHA, and the American Stroke Association [2,16]. A presidential advisory document from the AHA and the American Stroke Association has suggested that new and sustainable rural-specific and team-based care models assisted by technology may be a solution to improve the delivery of care in rural communities [2]. The question of this study was what are the characteristics of a rural-specific, team-based model for the delivery of care assisted by technology that is feasible in “real-world” rural clinics? The study goal was to develop and evaluate the feasibility of a new team-based model for rural practices with the following 2 components: a care model (the socio component) and a technological component (the clinical decision support [CDS] system).

It has been proposed that team-based care involves collaboration between physicians and nonphysician health professionals for the delivery of care instead of the traditional model in which care is delivered by physicians only [17]. A previous study showed that pharmacists working in collaboration with other health professionals in a team-based model improved cardiovascular health [18]. A second study demonstrated that a team-based delivery model using both physicians and advanced practice providers delivered outpatient cardiovascular care of a similar quality compared with a physician-only model [17,19].

For the sustainable adoption of new models for care delivery, it is fundamental that intended users are involved in the design process to ensure integration within redesigned user workflows [20,21]. Previous studies have demonstrated that well-executed participatory design processes support the implementation of health interventions [22-29]. According to Carrol and Rosson [30], participatory design advocates that users be included in the design process, and their input will increase the likelihood of successful design. According to Clemensen et al [31], the main feature of this design approach is the participation of users who work with researchers to produce new technology systems that can be understood and managed in practice.

Previously, in underserved rural settings, participatory design has informed strategies for the development of scalable systems such as mobile technology to disseminate health information for reproductive and child health services [32] and electronic, tablet-based community assessment tools for food and physical activity assessment [33]. When conducted under the sociotechnical theory framework, participatory design promotes the adoption of health care IT systems, including CDS [21,34]. The sociotechnical systems theory encourages the joint design of both the social and technical elements of a system [35]. A purely technocentric approach to system design may be unable to address the complex relationships between human and social factors and technology within the organizational context [36]. Therefore, in this study, participatory design under the sociotechnical system theory framework was used to design a new rural-specific and team-based care model for the coordination and delivery of secondary prevention to patients with ASCVDs assisted by CDS technology.

## Methods

### Setting and Context

The Office of Management and Budget defines rural counties as those with an urban core of ≥10,000 to <50,000 people [2]. By this definition, Austin, an urban core of 25,000 residents located in Mower County, Minnesota (MN), was identified as the site for the development of the rural-based model of care. Austin is the only urban core area in Mower County. The outpatient primary care clinic located in Austin where this study was conducted is part of the Mayo Clinic Health System (MCHS). The MCHS is a network of community-based health care professionals in primary care clinics located in >60 communities in MN, Iowa, and Wisconsin. Within this care network, patients receive primary care in their own communities. These clinics use the Mayo electronic health record (EHR) with digital medical data stored in a common centralized data warehouse that enables the deployment of CDS populated by EHR data for use in the rural clinics of the MCHS. Austin is located within driving distance (42 miles) of Rochester, MN. The research team drove to Austin or connected remotely with Austin providers and patients during this study. The design, IT, and clinical informatics teams were from Rochester, whereas the rural primary care teams that participated in the study worked in the MCHS Austin. Primary care providers in the primary care teams included physicians and advanced practice providers such as nurse practitioners (NPs) and physician assistants. Primary care nursing supported the day-to-day work of primary care providers within a team and included registered nurses (RNs) and licensed practical nurses (LPNs). The expanded primary care team supported multiple primary care teams and included pharmacists, tobacco cessation coaches, and other teams of nurses such as care coordinators and complex disease coordinators. Importantly, all teams collaborated in the design of a rural-specific and team-based model for the delivery of care from June 2019 to December 2020.

### Ethics Approval

This project was approved by the Mayo Clinic Institutional Review Board (approval numbers 19-011925 and 20-001192).

### Project Phases

#### Overview

**Figure 1:**
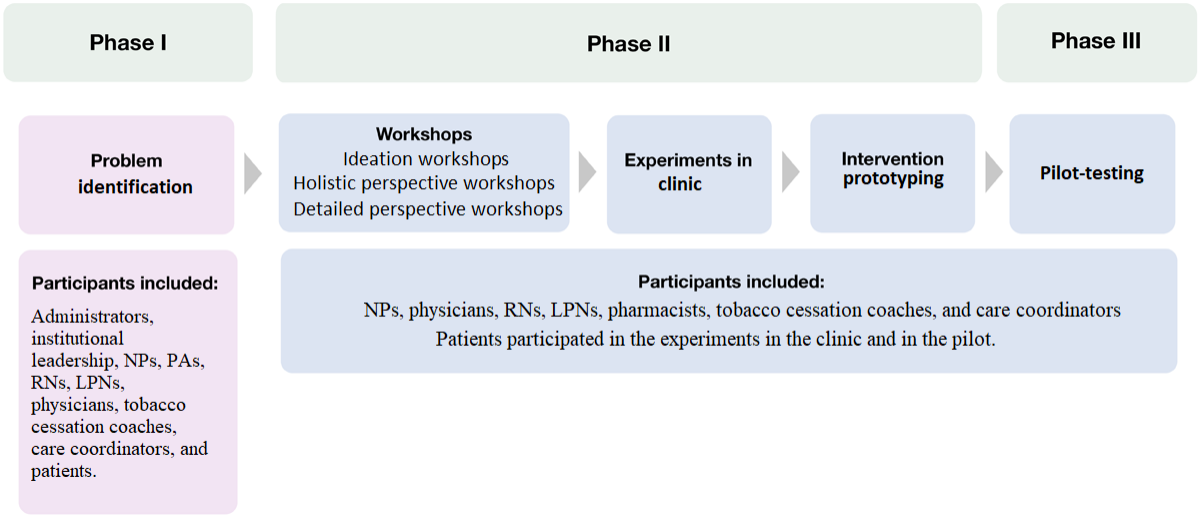
Project phased-design approach. LPN: licensed practical nurse; NP: nurse practitioner; PA: physician assistant; RN: registered nurse.

The project had three distinct phases: (1) problem identification, (2) experimentation, and (3) testing (Figure 1). The first aim of the activities in phase I was to gather information about the current status of ASCVD secondary prevention management. The second aim of this phase was to discuss ways of improving delivery of care to these patients. A cohesive group of participants was established representing different stakeholders (Figure 1). In phase II, the aim was to gain insights into possible solutions for the identified problems, creating and testing prototypes in experiments conducted in clinical practice. In phase III, the intervention prototype was tested and iteratively refined with user feedback in a 3-month pilot trial.

In this study, the participatory design activities of telling, making, and acting were conducted in iterative plan-action-observe-reflect cycles [31,37]. The provider workflows and CDS technology components of the sociotechnical model were developed using design activities and plan-action-observe-reflect cycles. The MCHS Austin leadership identified and recruited a team of local health care professionals to participate in this study. Patients who underwent medical visits in the MCHS Austin were recruited for interviews and observations. For the pilot trial, the patient cohort was identified electronically via a Cohort Knowledge Solution platform, and the patients were randomized into the control or intervention arm.

#### Phase I—Problem Identification

Phase I had 2 components: problem identification and ideation workshops.

##### Problem Identification

- Overview: The purpose of this phase was to gather data about the intervention context, including the setting, barriers, and facilitators of integration of the proposed technology solution into the rural clinic [31,37,38]. This phase focused on telling design activities. Applied ethnographic methods of participant observation and interviews comprised the telling activities [31,37], conducted during 75 hours of workflow observation in the clinic and 18 semistructured interviews with patients and health care professionals. Purposive sampling was used to identify interview participants with relevant roles and expertise, including administrators; institutional leadership; and various clinical health care professionals, including pharmacists, RNs, NPs, tobacco cessation coaches, and physicians. In this phase, 8 patients (mean age 77, SD 10 years; n=6, 75% women and n=2, 25% men) were also interviewed after primary care visits. Research team members (MEK, MP, and NP) performed rapid content analysis on observation field notes and verbatim interview transcriptions to identify key concepts and themes related to user needs for a sociotechnical-driven integration of the CDS technology [39]. The results of the analysis were synthesized to inform making and prototyping activities.
- Output from problem identification: The insights from problem identification that informed the design of the ideation workshops are outlined in Textbox 1.

**Insights from the problem identification component.**

**Insights from patients and providers**

- For many patients, especially those not meeting cardiovascular guideline (*V4*) recommendations, cardiovascular care was a low priority and, consequently, not addressed during office visits. Patients cited frequent provider turnover as a barrier, expressing uncertainty about who was responsible for managing cardiovascular health. The following are examples from patients:
  “It is hard to get an appointment.”
  “It isn’t like you can call in and see your doctor when you don’t feel right.”
  “I find it extremely difficult to see anyone.”
  “Our one thing here is, getting a doctor and keeping a doctor.”
- The cost of medications was not considered a barrier, but the cost of visits was a frequent concern. The following are examples from patients:
  “I have always had really good medical insurance that help cover the cost, it’s never been a problem.”
  “The cost of all this is just astronomical.”
- Patients who had previous intolerance for medications prescribed for cardiovascular prevention were reluctant to try another medication, especially in the absence of a relationship with a trusted provider. The following are examples from providers:
  “Patients with elevated LFTs so a barrier to statin therapies and using it comfortably in those types of patients.”
  “People who are not on statin who are have a cardiovascular event usually have problems with tolerating statins before so then it’s just sort of going down the statin.”
- Rural social networks are tightly knit, indicating that health care would ideally be delivered by local professionals. The following are examples from providers and patients:
  “You form that connection and they look to you, a familiar face” (provider).
  “People feel like they can trust us, that it’s a well-established practice” (provider).
  “After we meet them for the first time, we develop relationships, they can see we can help” (provider).
  “They become like family” (provider).
  “They rely on you” (provider).
  “Just knowing that you are going to be with them on their journey. They feel better about that” (provider).
  “It depends on how I feel about the person. If I trust them” (patient).
  “Feeling like you have someone’s undivided attention” (patient).
  “I think they want to work with us” (patient).

##### Ideation Workshops

The ideation workshop structure was as follows:

- Overview: The purpose of the ideation workshops was to discuss future ways of organizing the delivery of care for patients with ASCVDs. In total, 2 ideation workshops were conducted. Each workshop drew health care professionals from various roles, including 5 RNs, 4 NPs, 3 LPNs, 2 pharmacists, 3 physicians, 1 tobacco cessation coach, and 1 care coordinator.
- Output from the ideation workshops: The insights from the ideation workshops that informed the design of the phase II experimentation workshops are outlined in Textbox 2.

**Insights from the ideation workshops.**

**Insights from providers**

- Nonphysician care team members were motivated to collaboratively deliver preventive cardiovascular care to patients. However, there were no dedicated workflows and tools to support such initiatives. The following are examples from providers:
  “I don’t know sorting it out by their blood pressures, whether they’re elevated, just being on medication or not, obviously smokers, are they on medications? What meds?”
  “Being able to distinguish the groups like that might be helpful for us in determining where our resources should go.”
- Preventive cardiovascular care should be proactively and intentionally delivered. The following are examples from providers:
  “We don’t get referrals like we used to.”
  “They probably need a visit with the provider.”
  “Some of them definitely should have been seen by a provider just because of the length of time they’ve been seen.”
  “They’re not able to take that medication is there something else we can find for them.”
- Improving the delivery of preventive cardiovascular care on a systematic level cannot be regarded as a low priority. The following are examples from providers:
  “They just get through if they’ve been in the hospital.”
  “Identifying those patients that need more care and making sure that they are getting scheduled every so often, just for checking in, so that its keeping them out of the ED and out of the hospital.”
- Existing personnel should be dedicated to the intentional delivery of preventive cardiovascular health care. The following are examples from providers:
  “Regular appointments rather than waiting for something to happen...and they’re probably going to need more time then we can give in fifteen to thirty minute appointments.”
  “Get back into that role of relationship building and connecting with people and then from there we can then take the next step.”

#### Phase II—Experimentation

This phase had 3 components: prototyping workshops (including holistic and detailed perspective workshops), experiments in the clinic, and intervention prototyping.

##### Prototyping Workshops

The prototyping workshop structure was as follows:

- Overview: The purpose of the prototyping workshops was to design components of the novel model for the delivery of preventive cardiovascular health care for rural communities. The prototyping workshops involved making design activities. In making activities, user workshops were conducted to generate ideas to address issues identified during the telling activities and tailor the intervention to the needs of users and the context of the rural clinic. The multidisciplinary workshop methodology proposed by Scandurra et al [28] was used for the workshops. Prototyping workshops covered holistic and detailed perspectives from the different types of professionals on the care team [28].
- Holistic perspective workshops: There were 2 multidisciplinary interprofessional prototyping workshops in phase II. These workshops covered strategies for co-operation between different professionals [28] and included 4 RNs, 4 NPs, 3 LPNs, 2 pharmacists, 1 tobacco cessation coach, 1 RN care coordinator, and 3 physicians. In the first of these workshops, the staff suggested possible experiments. In the second workshop, participants selected the experiments and built on the proposed experiments in an iterative process. In these workshops, pamphlets summarizing insights and ideas were the discussion-inducing artifacts to facilitate collaborative and iterative idea generation [40].
- Detailed perspective workshops: These workshops included 1 health care professional work category each and focused on details of current and future professional workflows with discussion, feedback, and usability tests. There were workshops for nurses (4 NPs, 3 LPNs, 4 RNs, and 1 RN care coordinator), pharmacists (n=2), and tobacco cessation coaches (n=1). Artifacts for these workshops included system workflow diagrams, CDS user screenshots, deidentified patient information, drafts of templates for clinical notes summarizing encounters, and handouts summarizing insights from experiments.
- Output from prototyping workshops: Health care professionals participating in the prototyping workshops designed the “rooming reminder” and “reaching out” prototypes. Both prototypes were evaluated during experiments in the clinic.

##### Experiments in the Clinic

**Figure 2:**
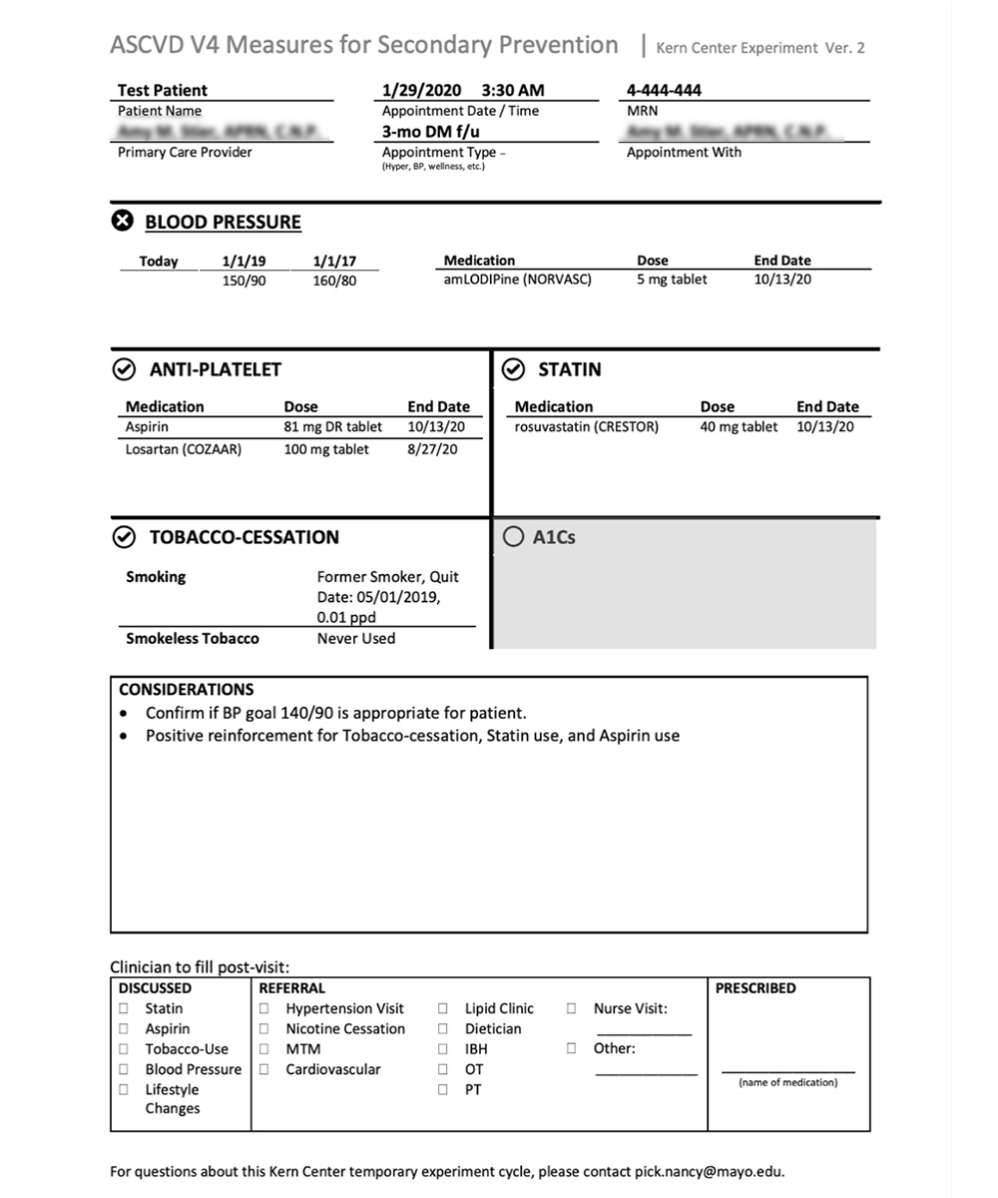
Cardiovascular-Patient Appointment Note handout summarizing the status of use of guideline recommendations by a patient with atherosclerotic cardiovascular disease. Handouts were given to primary care providers before encounters.

**Figure 3:**
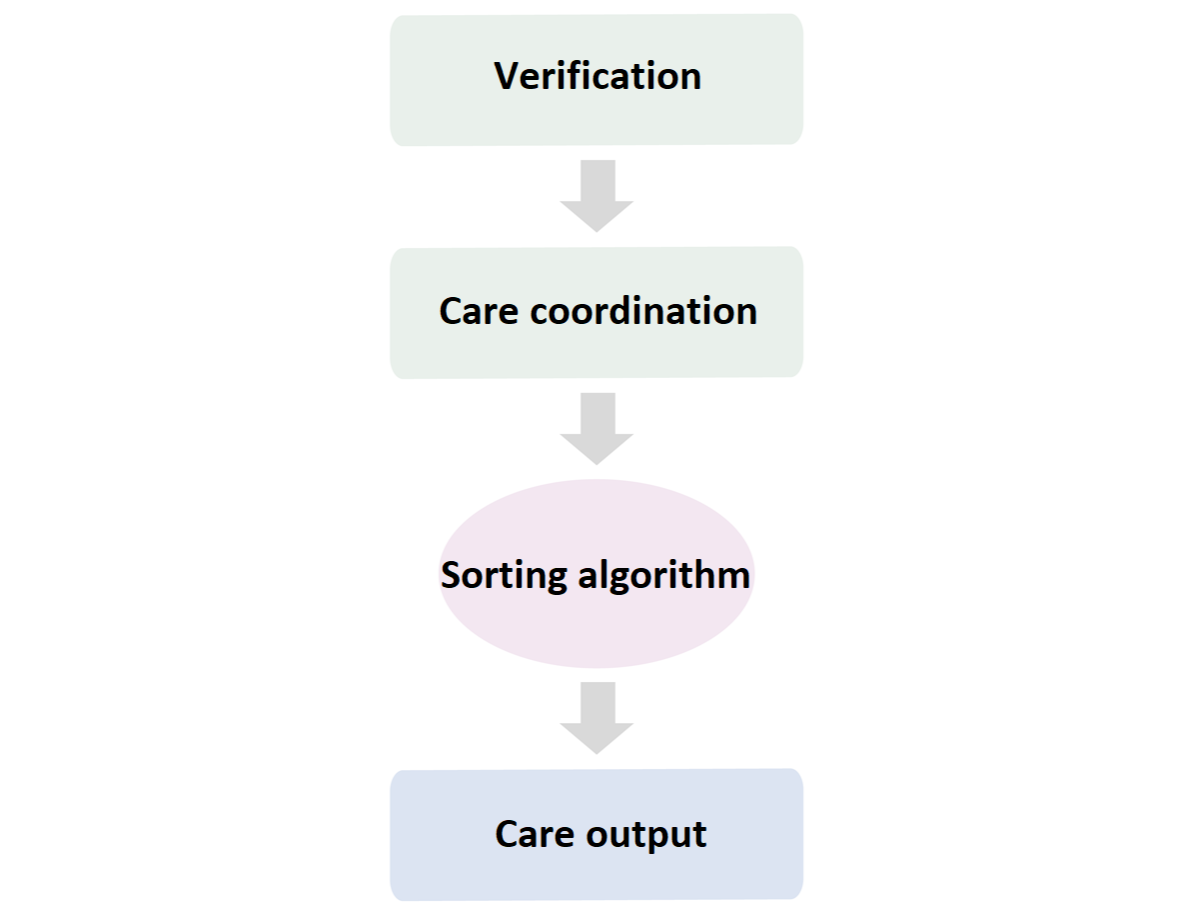
Design of the reaching out experiment.

The experiments in the clinic were carried out as follows:

- Overview: The purpose of the experiments in the clinic was to explore how designs affect and change practice. This phase focused on acting design activities. For acting, activities were conducted as prototype intervention experiments. The experiments enabled quick testing of the prototypes and evaluation of new ideas through iterative cycles [31,37]. During >37 hours of experimentation, 2 prototypes were evaluated. The experiments evaluated the “rooming reminder” and “reaching out” prototypes.
- Rooming reminder experiment:
  Overview: Insights from patients and health care professionals indicated that, often during routine medical encounters, other complex medical issues are prioritized, and cardiovascular prevention is not addressed. These insights led to the decision to design a rooming reminder experiment. The purpose of the rooming reminder was to remind clinicians to address cardiovascular prevention during upcoming encounters using handouts that summarize the gaps in preventive cardiovascular care for each patient. Handouts were created and named the “Cardiovascular-Patient Appointment Note” (Figure 2). During the experiment, hard copies of these handouts were given to primary care providers by a desk attendant for rapid review before the encounter. The design team observed the Cardiovascular-Patient Appointment Note impact on provider-patient interactions and the content of the medical visits. After 5 days, the team concluded that the Cardiovascular-Patient Appointment Notes had minimal impact. The notes affected only 21% (3/14) of the visits from patients with ASCVDs from a total of 196 visits during this time frame. Providers were interviewed before starting the experiment and asked follow-up questions after experiment completion. In addition, providers were observed during the rooming reminder experiment.
  Output from the rooming reminder experiment: In total, 3 main insights were gained from the rooming reminder experiment. First, not all the information in the EHR was up to date. Second, only a small number of patients not meeting V4 metrics came to the clinic weekly, suggesting that focusing on current in-visit care will not have the greatest impact. Third, the experiment made clear that the visit context was a major influence on whether cardiovascular health was evaluated. Clinicians used their judgment to determine whether the visit context was appropriate to discuss individual patient cardiovascular metrics. The Cardiovascular-Patient Appointment Note successfully prompted cardiovascular health conversations when all variables identified in Textbox 3 were met, which was rare. Although prompting discussions on cardiovascular prevention during routine outpatient visits can affect care, there is more opportunity to optimize community health through intentional encounters focused on cardiovascular prevention.

- Reaching out experiment:
  Overview: Insights that guided the “reaching out” experiment were that cardiovascular prevention is often not addressed during routine medical encounters and that rooming reminders had minimal impact. The purpose of the reaching out experiment was to actively contact patients for intentional delivery of preventive cardiovascular care. This experiment had three phases: (1) verification, (2) care coordination and sorting algorithm, and (3) care output (Figure 3). In the reaching out experiment, a total of 8 workflows and 48 processes were developed and tested. A detailed description of these 3 phases is provided in the following sections.
  Verification phase: Overview: We learned that clinicians must trust the information used for patient management. However, EHR information is often outdated and should be verified with patients before making decisions. The purpose of the verification phase was to gather updated information on the use of guideline-recommended strategies directly from the patients. Output from the verification phase: The initial verification had 2 stages. First, messages were sent through the Mayo Clinic portal app containing a survey asking patients about their cardiovascular prevention status. A team member called patients who did not reply to portal messages and conducted a scripted telephone interview with the same questions used in the survey sent via the portal app. Textbox 4 shows the patient survey questions. As an initial proof of concept, 89 web-based surveys were sent to patients active on the Mayo Clinic portal app. The response rate was 40% (36/89).

- Care coordination and sorting algorithm phase:
  Overview: The purpose of this phase was to define criteria to assign the right patient to the right provider. The insight that informed this step was the need to assign the right patient to the right provider. Provider skill sets had to match patient gaps in preventive care such that health care professionals with the appropriate skill set would be assigned to evaluate patients with specific gaps in preventive cardiovascular care. Examples from providers are shown in Textbox 5.
  Output from the care coordination and sorting algorithm phase: Initially, health care professionals (2 pharmacists, 2 RNs, and 1 advanced practice provider) reviewed the charts of 10 patients with ASCVDs and recommended which care team role should be assigned to each patient. Subsequently, the design team worked with health care professionals to articulate criteria for an automated sorting algorithm assigning patients to the most appropriate team member (both in terms of licensure and specialty) to deliver care plans to each patient tailored to care gaps.
- Care output phase:
  Overview: The purpose of “care output” was to use patient preference for the selection of the type of encounter for the delivery of cardiovascular prevention by rural providers. The insight that informed the “care output” was that patient preference defined the type of encounter for the delivery of preventive cardiovascular care. The options for encounter types were phone call conversation, telemedicine, or in-person visit. For example, patients with limited access to transportation may prefer either phone call conversations or telemedicine visits. By contrast, those with access to transportation may prefer an in-person visit. Examples from providers and patients are shown in Textbox 6.

- Output from the care output phase: This experiment created and tested workflows to assign encounter types based on patient preference and 10 templates for clinical notes documenting encounters.

**Factors needed before initiating a cardiovascular preventive health conversation with patients.**

**Factors necessary for starting cardiovascular prevention evaluation**

- Visit appropriateness: visits regarding well-managed chronic care more readily transitioned to a conversation on cardiovascular metrics. By contrast, complex or uncontrolled comorbidities became top visit priority and left little time for other discussion.
- Patient appropriateness: provider perception of patient workload to capacity determined whether the provider would address cardiovascular metrics.
- Provider priorities: providers addressed what they viewed as patients’ health priorities. Providers needed to believe cardiovascular guideline (*V4*) recommendations were important for patient health for them to address them. Trust between patient and clinician further enabled dialogue and negotiation related to cardiovascular care.
- Trust in information: providers needed to believe that the recommendations were tailored to each patient case and not based on generic guidelines.
- Actionability of information: actionable recommendations reduced the time required for providers to match up information with next steps for patients.

**Questions in the patient verification survey.**

**Questions**

- Do you currently take a daily aspirin?
- Do you know the dose of aspirin you take daily?
- Do you know another antiplatelet medication you take? Which dose?
- Do you know which statin you take? Which dose?
- Have you tried a statin in the past? Did you experience any adverse reactions because of the statin you took previously?
- Would you be open to our team’s medication expert connecting with you to discuss medication strategies for reducing your risk of future cardiovascular episodes?
- Are you taking daily blood pressure (BP) medications?
- Would you be open to a care team member (a pharmacist or nurses) working with you on custom strategies to lower your BP?
- Do you have a BP monitoring device at home?
- If known, what was your last BP reading from your home BP monitor? What date was it taken?
- Are you currently using tobacco products?
- Would you be open to our tobacco cessation coach calling you to offer information or see if you have questions?
- If your care team wishes to recommend next steps, what would be the best means to contact you? Messages via the Mayo app, phone call, or both?

**Example quotes from providers for the care coordination and sorting algorithm phase.**

**Example quotes**

- “The only thing about blood pressure vs statin and aspirin, statin and aspirin are you, you’re on it or you’re not on it, that’s it, blood pressure we’re adjusting and fine tuning.”
- “Smoking is definitely its own thing.”
- “A lot of different medications, there are certain ones that need lab work, how often am I going to need to monitor you.”
- “Statin and aspirin at a certain level you’re prescribing it now in a perfect world they are taking it as well. But you’re prescribing it whereas smoking and blood pressure are contingent on patient behavior.”
- “They need different tracks.”
- “Different providers own different groups.”

**Example quotes from providers and patients for the care output phase.**

**Example quotes from providers**

- “Assisted living or at home, are they home bound?”
- “Can they get out?”
- “Barriers to even getting here (at the clinic) in the first place.”
- “Where people live and how they get here (at the clinic).”

**Example quotes from patients**

- “See somebody [health care provider] personally, I prefer that.”
- “I like the good old phone call.”
- “Coming to the clinic, oh it’s nice to be out.”

## Results

### Intervention Prototyping

#### Overview

The purpose of this phase was to iteratively design an intervention prototype with health care professionals based on the insights from the experimentation phase. The design team delivered handouts for health care professionals during detailed perspective workshops summarizing insights from phase II experiments to facilitate collaborative and iterative idea generation [40].

**Figure 4:**
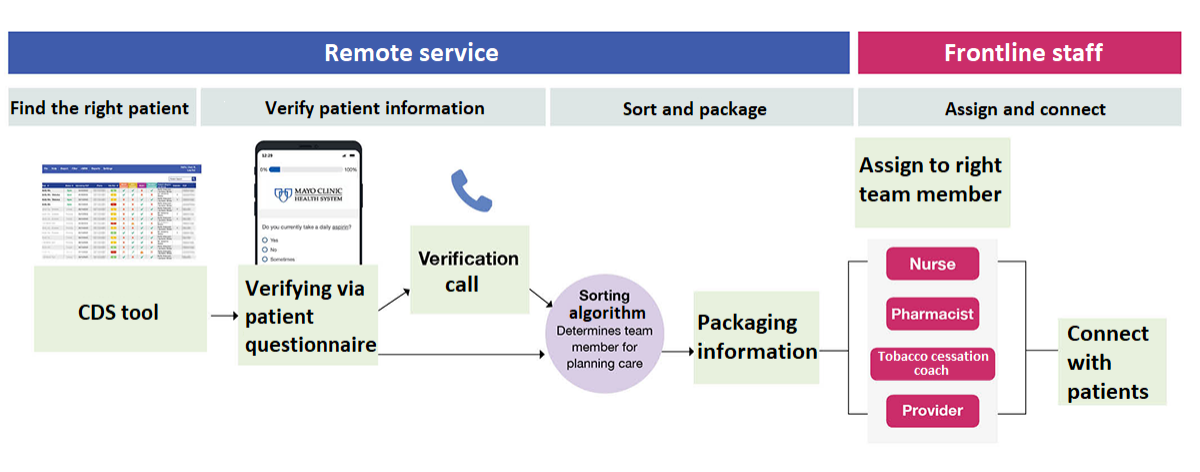
The final resulting intervention design is a sociotechnical system—a combination of roles, processes, and technology—to enable primary care teams to improve the delivery of cardiovascular prevention strategies. CDS: clinical decision support.

This process resulted in the design of a sociotechnical intervention prototype with the following components: (1) finding the right patient, (2) verifying patient information, (3) sorting and packaging patient information, and (4) assigning the right provider and connecting with patients (Figure 4). Each component of this intervention is described separately in the following sections.

#### Finding the Right Patient

On the basis of stakeholder feedback, rapid prototyping of the CDS tool was conducted in the testing environment of the web-based technology platform, termed Cohort Knowledge Solution, using the Agile Scrum methodology for software development [41]. The Cohort Knowledge Solution platform is populated by EHR data that are computationally extracted from the institutional data warehouse. Computational phenotyping algorithms were installed in the Cohort Knowledge Solution to automatically identify patients with ASCVDs and identify individual gaps in adherence to *V4* recommendations [42].

ASCVDs include coronary artery disease, peripheral artery disease (PAD), and ischemic stroke [43]. Rule-based billing code algorithms for identification of patients with these conditions were deployed via the Cohort Knowledge Solution platform. Random samples of the retrieved data were manually reviewed by a trained abstractor following the written criteria for standardization to create a reference standard. The processes to support accurate data collection were developed using iterative validation cycles and used Boolean combinations of billing codes [44]. The rule for retrieval of coronary artery disease was a diagnostic code for myocardial infarction or angina pectoris or a procedural code for coronary revascularization procedure (percutaneous or surgical); for ischemic stroke, it required the presence of an International Classification of Diseases, 10th Revision, diagnostic code for ischemic stroke or transient ischemic attack; and, for PAD, a diagnostic code and International Classification of Diseases, 10th Revision, procedural code for lower extremity limb revascularization (endovascular or surgical) was required. Performance metrics were generated by comparison with the reference standards (Table 1).

Given the inferior performance of billing codes for PAD cohort identification, a natural language processing algorithm for the extraction of PAD from clinical narratives was also created and validated with a sensitivity of 91% and a positive predictive value of 92% [45]. This previously validated natural language processing PAD algorithm was also deployed to the Cohort Knowledge Solution to identify cases.

**Figure 5:**
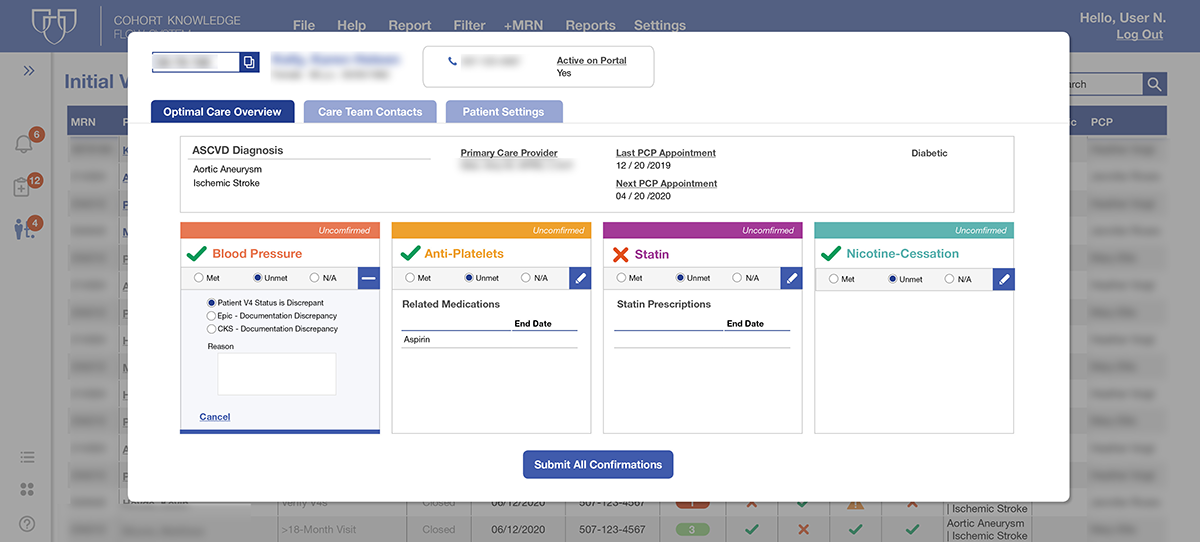
Cohort Knowledge Solution platform redesigned after user testing with health care professionals.

Wireframe usability tests were conducted with care team members to identify which data were most relevant for planning and delivering preventive cardiovascular care. The technology developed also enabled these data to be retrieved from the EHR data and displayed on the same screen with a single mouse click (Figure 5).

**Table 1:** Performance metrics for billing code algorithms in the Cohort Knowledge Solution.

Type of ASCVD^a^	Charts reviewed, N	Sensitivity, %	Positive predictive value, %	F_1_ score, %
Coronary artery disease	189	96	94	95
Peripheral artery disease	140	64	100	78
Stroke	156	98	81	89

^a^ASCVD: atherosclerotic cardiovascular disease.

#### Verifying Patient Information

**Figure 6:**
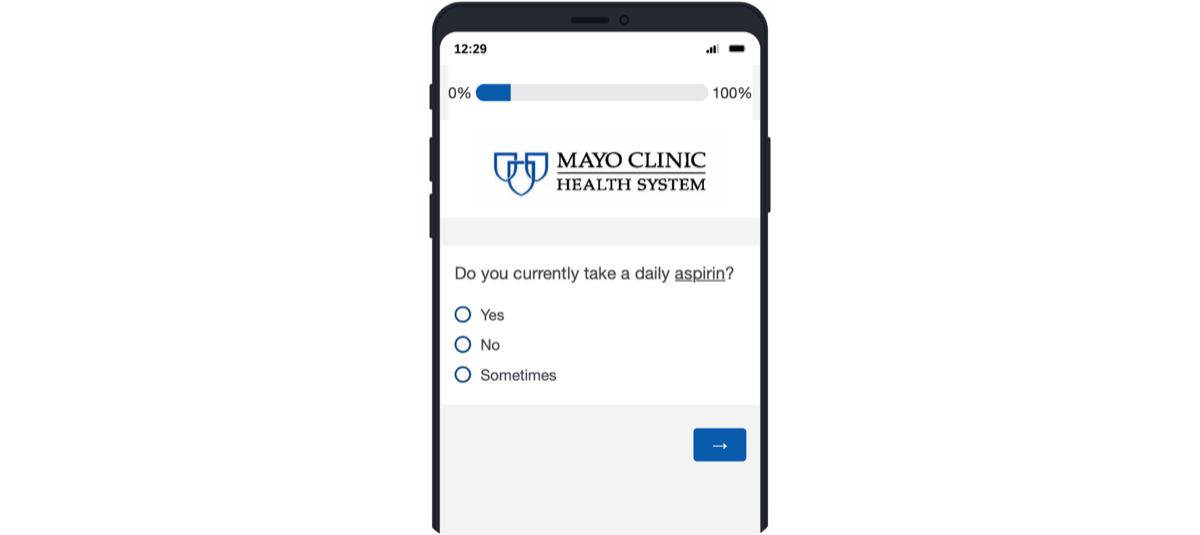
Screenshot of the verification survey sent via the Mayo Clinic patient portal app.

Health care professionals need the right information to deliver medical care efficiently and effectively. Messages were delivered to patients through the Mayo Clinic portal app with survey questions regarding adherence to *V4* recommendations (Textbox 4 and Figure 6). Patients who did not complete the portal survey, did not answer 3 phone calls from the study team, or declined to participate in this project continued to receive care through the usual model of care. Patients who opted to participate in the intervention were connected with health care professionals with expertise tailored to patient gaps for cardiovascular prevention management. Survey responses were used to update the EHR of each patient, and workflows were designed to support the verification process.

#### Sorting Algorithm

The design team adapted the sorting algorithm into criteria that the informatics team translated into a sorting computational algorithm for the Cohort Knowledge Solution. Examples of criteria included “if patients were missing statin or anti-platelet, send to pharmacist” or “if a patient reported taking an undocumented anti-platelet, message nurses to update the medical record.” The workflows were redesigned by incorporating the new CDS technology.

#### Packaging Patient Information

The design team originally assumed that the local care team members (eg, pharmacists and nurses) would use the platform. However, during usability testing, the participant health care professionals expressed dissatisfaction with the thought of using another tool for clinical practice. Accordingly, the workflow was redesigned to have a dedicated remote user—analogous to the role of an “air traffic controller.” This dedicated remote user took responsibility for care coordination using the Cohort Knowledge Solution in a central hub model. Once patients had filled the verification questionnaire, the dedicated user aggregated relevant patient information within the Cohort Knowledge Solution and electronically assigned it to the right provider.

#### Assigning the Right Provider

The dedicated user leveraged the Cohort Knowledge Solution sorting algorithm to identify the right providers and sent an in-basket message with the aggregated medical information. The care team members, often nonphysician health care professionals, used the packaged medical information to plan care and “connect with patients” following workflows specifically designed for this phase.

#### Connecting With Patients

Rather than developing new cardiovascular visits, we focused on routing patients to the existing visit types. Therefore, the redesigned workflows assigned the right patient to the right provider. The provider reviewed the packaged medical information and initiated communication, such as phone calls, video telemedicine, or face-to-face encounters, based on patient preferences and needs.

#### Centralized Hub Model

**Figure 7:**
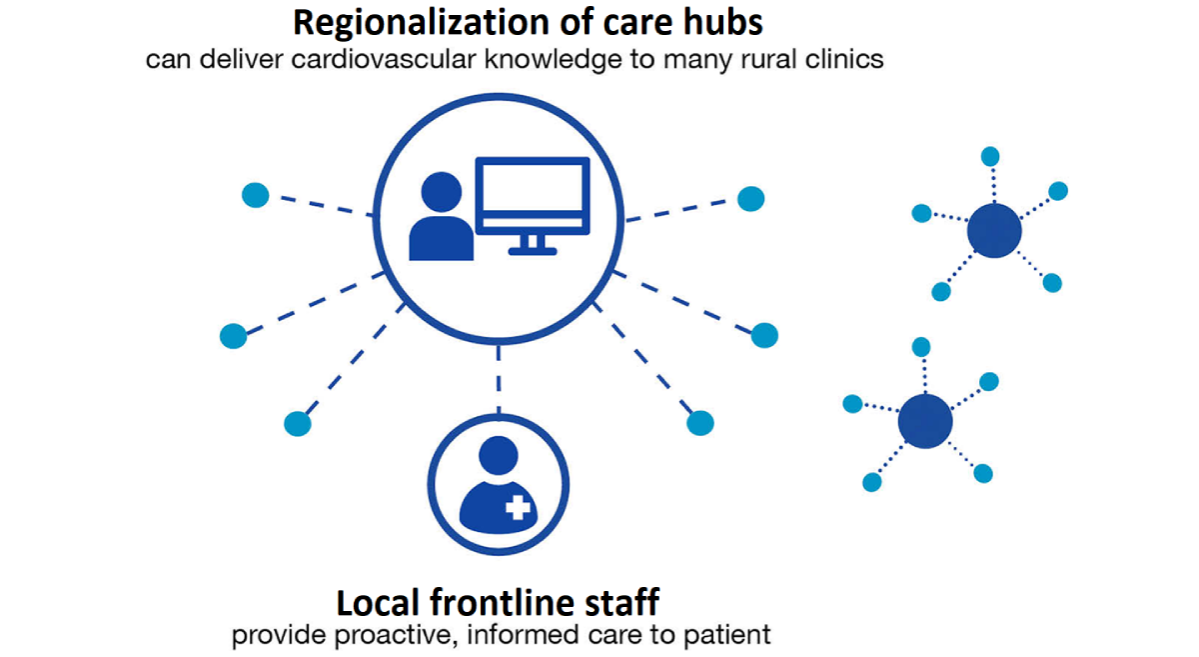
The regionalization of the care hub model.

To address obstacles stemming from the rural provider shortage, a regionalization of the care hub model was designed (Figure 7). Verifying and packaging patient information took place in a central rural hub with dedicated resources assisted by technology to gather and summarize the patient-specific gaps in preventive care. The information gathered at the central hub was shared with the right rural provider for delivery of care locally. The central hub gathered all the information necessary for the delivery of care, reducing the need for manual chart review by health care professionals and enabling them to focus on delivering tailored care.

### Phase III—Testing

A 3-month prospective randomized pilot trial was conducted in the outpatient primary care clinic in Austin, MN. The goal of this pilot was to test and iteratively refine the sociotechnical intervention prototype with all the components, as shown in Figure 4. Patients with ASCVDs were assigned to the intervention or control arm by stratified randomization with strata based on the number and types of *V4* cardiovascular recommendations in use. The intervention was a rural-specific and sociotechnical model prototype that was proactive and delivered preventive cardiovascular care specific to each patient. The care delivery model for the control arm was usual care, which is a symptom-based and reactive model. In the usual care model, health care professionals rely on patients to contact the system for evaluation of symptoms [46,47]. Patients assigned to the control and intervention arms were evaluated during the same period. A Cohort Knowledge Solution ASCVD registry was used to identify patients with ASCVDs. A total of 369 patients with ASCVDs on ≤3 *V4* guideline recommendations were randomly assigned to the control (n=182, 49.3%) or intervention (n=187, 50.7%) arm. Baseline clinical characteristics were similar in patients assigned to the 2 study arms (Table 2).

**Figure 8:**
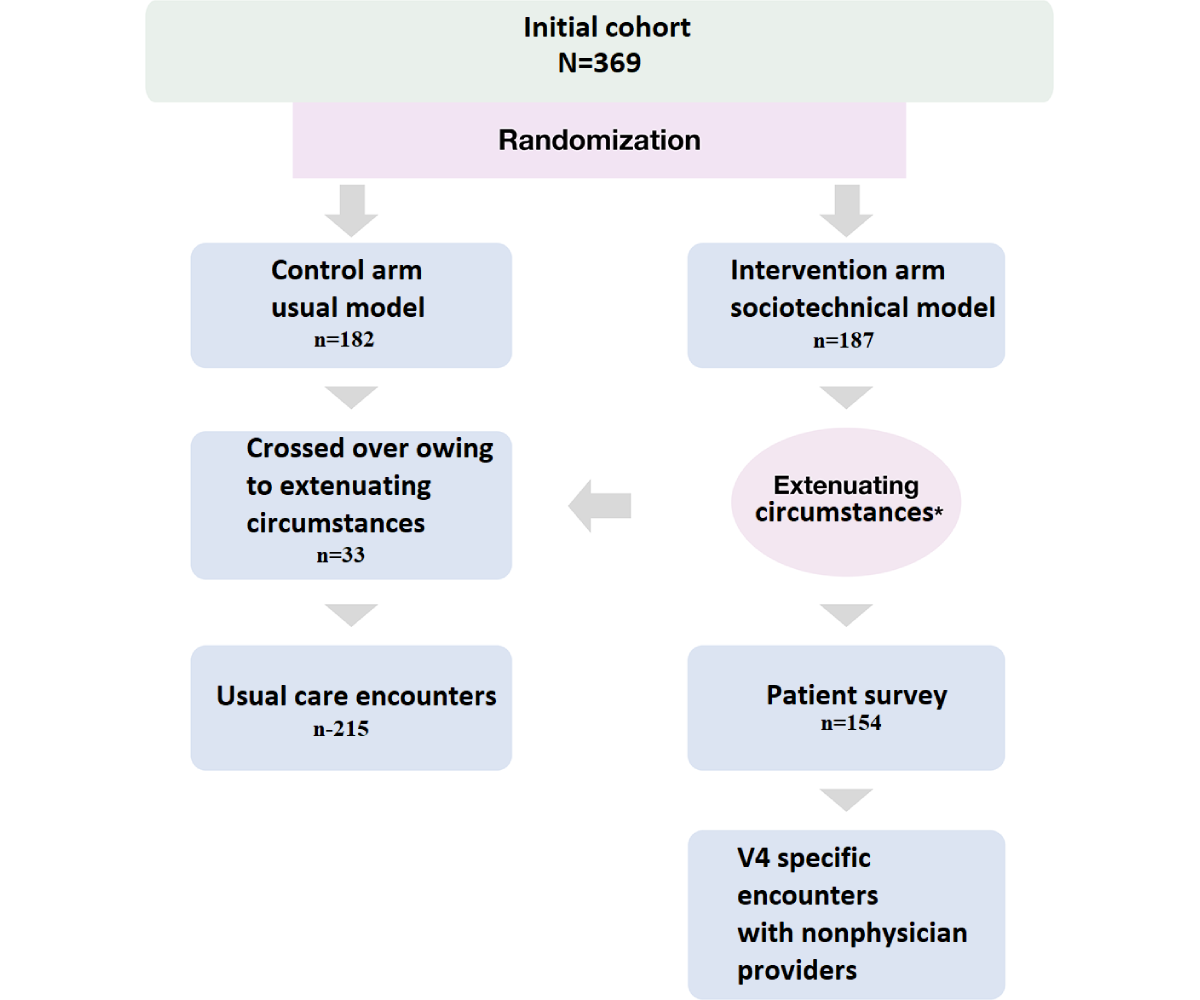
Pilot trial design. V4: cardiovascular guideline recommendations. *Extenuating circumstances included dementia or cognitive impairment, end-stage medical conditions on hospice care, active cancer treatment, hospitalization during the pilot, patient relocation to a different county and no longer receiving medical care from the Mayo Clinic Health System Austin, upcoming cardiology appointment within 3 months of the pilot, or other medical conditions that did not require the use of V4.

Subsequently, the medical records of all patients were reviewed by a trained abstractor before surveying to find extenuating circumstances that would justify not reaching out to a given patient during the pilot. These circumstances included dementia or cognitive impairment, end-stage medical conditions on hospice care, active cancer treatment, hospitalization during the pilot, patient relocation to a different county and no longer receiving medical care from MCHS Austin, upcoming cardiology appointment within 3 months of the pilot, or other medical conditions that did not require the use of *V4* (Figure 8). These reasons were discussed with health care professionals in the detailed perspective workshops and subsequently incorporated into the workflows.

A total of 33 patients crossed over to the usual model of care arm for analysis after this review. After crossing over, 82.4% (154/187) of eligible patients remained in the intervention arm and were surveyed. The patient survey (depicted in Textbox 4) was a component of the intervention and was delivered via portal messages or addressed during scripted telephone interviews. Patients in the intervention arm completed the verification survey and answered the following question: “If your care team wishes to recommend next steps, what would be the best means to contact you? Messages via the Mayo app, phone call, or both?” The patients were contacted using their preferred strategy. Those who did not respond to this question were not contacted. Of the 154 contacted patients, 86 (55.8%) responded to the patient survey via electronic messages or scripted telephone interviews in <3 months. These patients were subsequently assigned to nonphysician health care professionals for the proactive delivery of patient-specific V4 strategies for secondary prevention. There were no differences in the proportion of patients implementing guideline recommendations between those who answered the survey via the patient portal and those who answered the survey by phone call.

The primary outcome of the pilot trial was the proportion of *V4* recommendations delivered, as measured by encounters with nonphysician health care professionals. Encounters with nonphysician health care professionals (nurses, pharmacists, or tobacco cessation coaches) included consults (in person or via telemedicine) or phone conversations. During the pilot, the proportion of patients who had encounters with nonphysician health care professionals for delivery of V4 recommendations in the intervention arm was greater for all types of professionals than in the control arm (Table 3). After exclusion of the 33 patients who crossed over from the intervention arm to the control arm, similar results were observed. In subsequent analysis removing all participants in the control arm who met the criteria for exclusion from the intervention arm (73/369, 19.8%), the results remained unchanged.

During and after the pilot, health care professionals were interviewed, and the information gathered was used to further refine the intervention. The pilot trial demonstrated that this model (1) connected the right health care professional with the right patient, (2) saved time by reducing the need for manual chart review, (3) enabled health care professionals to work to the top of licensure, (4) has potential for expansion to other conditions, and (5) promoted interdisciplinary collaboration to optimize care.

**Table 2:** Characteristics of patients assigned to the control and intervention arms (N=369).

Clinical variables^a^	Control arm (n=182)	Intervention arm (n=187)
Age (years), mean (SD)	71 (13)	71 (14)
Sex (male), n (%)	92 (50.5)	95 (50.8)
Race (White), n (%)	167 (91.8)	174 (93)
Ethnicity (“not Hispanic or Latino”), n (%)	171 (94)	178 (95.2)
Married, n (%)	94 (51.6)	103 (55.1)
Taking antiplatelet medications, n (%)	120 (65.9)	121 (64.7)
Taking statin medications, n (%)	111 (61)	112 (59.9)
Nonsmokers, n (%)	132 (72.5)	135 (72.2)
Blood pressure at goal, n (%)	87 (47.8)	89 (47.6)

^a^Two-sample 2-tailed *t* tests were used to compare means, and chi-square tests were used for comparison of percentages; all *P* values comparing the control and intervention arms were not significant (*P*>.05).

**Table 3:** Number of encounters for the delivery of cardiovascular guideline recommendations (V4) by nonphysician health care professionals during the pilot (after crossover; N=369).

	Control arm (n=215)^a^, n (%)	Intervention arm (n=154)^a^, n (%)	*P* value^b^
Nursing encounters for the delivery of *V4* recommendations	48 (22.3)	51 (33.1)	.02
Pharmacist encounters for the delivery of *V4* recommendations	1 (0.5)	31 (20.1)	<.001
Tobacco cessation coach encounters for the delivery of tobacco discontinuation strategies	3 (1.4)	30 (19.5)	<.001

^a^Total number of patients in each arm after crossing over.

^b^Two-sample 2-tailed *t* tests were used to compare means, and chi-square tests were used for comparison of percentages; for all analyses, *P*<.05 was considered significant.

## Discussion

### Principal Findings

This study used participatory design and the sociotechnical theory framework to create a team-based care model for the coordination and delivery of secondary prevention to rural patients with ASCVDs by nonphysician health care professionals. The new model of care redesigned workflows, integrated health care professional roles, and deployed a novel CDS technology. The subsequent pilot trial demonstrated the feasibility for effective implementation of this new model of care in a rural outpatient clinic. For the next phase of this project, a scalable intervention is planned to be implemented in other rural sites of the MCHS and evaluated in a multisite trial.

The requirements for successful practice transformation include changes in both workflows and technology [48]. Importantly, the optimal use of technology is dependent on the interrelation of the system with skilled and pragmatic work by health care professionals [49]. The design of systems focusing only on technological factors has been a major contributor to their underuse [36,50]. A previous study showed that, for most patients (80% of 5568 patients), providers disregarded the recommendations of a CDS to improve the use of ASCVD secondary prevention at hospital discharge [51]. In that study, the CDS was not part of a sociotechnical system and was not integrated with provider workflows [51]. The low use rate of an EHR-based CDS for cardiovascular risk reduction in community health centers was also reported in a cluster randomized clinical trial that focused on CDS technology [52,53].

This study reported a practical application of the sociotechnical system theory framework to design an intervention to improve the delivery of cardiovascular prevention. The sociotechnical design approach considers both technological and social factors to inform system design [50,54,55]. The use of a sociotechnical theory approach for system design leads to systems that are more acceptable to users and have long-term sustainability [36,56,57]. For these reasons, the sociotechnical design approach was used to design an intervention that will be likely sustainable when implemented in the rural sites of a large medical enterprise.

A previous study in India developed a CDS tool for cardiovascular risk screening used for single-visit encounters [58]. In that study, the CDS was not linked to information from EHRs, and it was not possible to follow patients longitudinally, which is a fundamental requirement for the workflow of providers managing secondary prevention strategies for patients with ASCVDs. By contrast, the CDS of this study was populated with data automatically extracted from EHRs, enabled the delivery of longitudinal care for patients with ASCVDs, and was integrated with provider workflows. A second study reported a CDS linked to the EHR for the primary prevention of patients without established ASCVDs in an urban setting, and a printed copy of the CDS summary screen containing the patient-specific status of use of guideline recommendations was placed on the exam door for rapid review by providers before the encounter [59]. In this study, the rooming reminder experiment used a similar approach. However, this strategy had minimal impact as not all the information in the EHR was up to date, only a small number of patients not meeting V4 metrics came to the clinic weekly, and the visit context had a major influence on whether cardiovascular health was evaluated. On the basis of these insights, the subsequent experiment (reaching out) aimed to actively contact patients for the intentional delivery of preventive cardiovascular care. Insights from the reaching out experiment became a core component of the new model for the intentional delivery of care designed for this study.

Health care professionals work in teams and are commonly assisted by computerized information systems. These systems display the information that different health care professionals need to complete their work. Scandurra et al [28,60] proposed multidisciplinary thematic workshops based on participatory design and computer-supported participatory work theories. This method uses a collaborative design and enables the translation of health care professionals’ needs into technical requirements. This study applied this methodology for the development of a team-based model of care, enabling multidisciplinary co-operation among team members for the delivery of preventive care for rural patients with ASCVDs.

A systematic review of the literature by Hardy et al [61] showed that access to the internet, digital literacy, and computer skills are important characteristics for the design of sustainable technology tools for residents of rural areas. This pilot recruited older adult patients from rural communities. Patient recruitment was first conducted using patient portal messages. We observed a low response rate to portal messages and, in the intervention group, 39.6% (61/154) did not have active portal accounts. Other studies have also demonstrated that access to and ability to use technology and the internet are barriers to the use of portals by older adult patients [62-64]. To overcome these barriers, in this study, patients who did not have active portal accounts or did not respond to portal messages were contacted by phone. The same survey questions sent via portal messages were used for scripted telephone conversations. The survey questions were simple and focused on patient-specific gaps in preventive cardiovascular care. The survey response rate of 55.8% (86/154) was superior to the 43.9% response rate of a previous survey of community-dwelling older adults in rural areas [14].

On the basis of our observations, rural providers need to consider patient preference for visit type to mitigate barriers to transportation and limited mobility, which often occur in rural residents, especially older adults, who were a major target group for this intervention. Compared with their urban counterparts, rural citizens are more prone to mobility and transportation barriers that make access to health care difficult [61]. In mitigation, phone conversations and telemedicine became options of visit types connecting patients and providers for the delivery of secondary prevention strategies for patients with ASCVDs. In addition, it is important to underscore that rural society values “neighborliness,” which manifests as trust in community members and potential distrust of outsiders [61]. The intervention developed in this study leveraged neighborliness and supported a rural place-based identity. Consequently, the hub model was maintained regionally rather than centrally in Rochester, MN (Mayo Clinic headquarters). The regionalization of the care model promoted the delivery of care by rural providers. During the implementation of this intervention, staff resources will be distributed to serve small rural clinics located in the area covered by a hub. In other rural health care settings, a similar process for the allocation of rural providers may be used.

The novel model of care described in this study assigns the right patient to the right professional for the delivery of preventive care. In addition, the selection of the encounter type was based on patient preference for in-person visits at the clinic, phone conversations, or video encounters via telemedicine. This flexibility facilitated access to health care professionals for patients with limited transportation resources. Furthermore, this model also enabled a patient-centered health connection that goes beyond traditional symptom-based visits. The primary outcome of the pilot trial was the proportion of *V4* recommendations delivered, as measured by encounters with nonphysician health care professionals. These metrics were set a priori following good practice. During the pilot, the proportion of patients who had encounters with nonphysician health care professionals for the delivery of *V4* recommendations in the intervention arm was greater for all types of professionals than in the control arm. Therefore, the pilot trial showed that this model connected the right health care professional with the right patient for the delivery of guideline-recommended strategies for patients with ASCVDs, demonstrating the feasibility of the intervention.

### Limitations

The pilot study was not powered to evaluate the differences in the use of specific guideline-recommended strategies. However, we are planning a subsequent prospective randomized implementation trial in the Midwest sites of the MCHS with central hubs spanning multiple rural clinics. This trial will be powered to evaluate the impact of the intervention on the proportion of patients using specific guideline-recommended strategies. In preparation for this implementation, the informatics and IT teams have been building the additional Cohort Knowledge Solution functionalities designed in this study, which are aligned with the new workflows. These functionalities include automation of the sorting algorithm and automated retrieval of extenuating circumstances.

The reason for not including patients in the workshops was that the Cardiovascular-Patient Appointment Note experiment showed that a major obstacle for the delivery of preventive cardiovascular care was the difficulty in reaching out to patients. Strategies for health care professionals to reach out to patients were developed to overcome this barrier. The cost of visits was a concern for patients. However, during this study, there was no additional cost for the patients. Further analysis of the cost of health care and strategies for billing will be performed during the implementation phase. During the pilot trial, health care professionals were asked questions about their experience with the intervention. However, patients were not asked similar questions. During the planned implementation trial, patients will be asked to identify barriers to and facilitators of the implementation of this sociotechnical intervention.

This novel team-based model of care was specifically designed to enable the delivery of care in resource-constrained clinics located in rural communities and promotes teamwork with shared responsibilities among team members. For clinics that do not have certain types of health care professionals (eg, pharmacists), we propose to use the resources of the regional hubs, in which health care professionals from other rural clinics could remotely support the teams where this specific expertise is not available. In addition, during the process of implementation, further information will be gathered about the characteristics of the intervention that are necessary for adaptation to other enterprises. Other implementation studies are needed to evaluate the reproducibility and scalability of this model of care to other enterprises that deliver health care to patients in rural communities.

### Conclusions

Participatory design within the sociotechnical theory framework enabled the development of a rural-specific, team-based care intervention assisted by CDS technology for the transformation of preventive health care delivery in rural clinics for patients with ASCVDs. By systematically promoting preventive care, this intervention has the potential to strengthen longitudinal relationships between clinics and their communities—the underlying requirement for secondary prevention for patients with ASCVDs in rural settings.

## Abbreviations

AHA: American Heart Association
ASCVD: atherosclerotic cardiovascular disease
CDS: clinical decision support
EHR: electronic health record
LPN: licensed practical nurse
MCHS: Mayo Clinic Health System
MN: Minnesota
NP: nurse practitioner
PAD: peripheral artery disease
RN: registered nurse
V4: cardiovascular guideline recommendations
